# The C-H Peripheral Stalk Base: A Novel Component in V_1_-ATPase Assembly

**DOI:** 10.1371/journal.pone.0012588

**Published:** 2010-09-03

**Authors:** Zacariah L. Hildenbrand, Sudheer K. Molugu, Daniela Stock, Ricardo A. Bernal

**Affiliations:** 1 Department of Chemistry, University of Texas at El Paso, El Paso, Texas, United States of America; 2 Victor Chang Cardiac Research Institute, Darlinghurst, New South Wales, Australia; 3 Faculty of Medicine, University of New South Wales, Sydney, New South Wales, Australia; University of Helsinki, Finland

## Abstract

Vacuolar ATPases (V-ATPases) are molecular machines responsible for creating electrochemical gradients and preserving pH-dependent cellular compartments by way of proton translocation across the membrane. V-ATPases employ a dynamic rotary mechanism that is driven by ATP hydrolysis and the central rotor stalk. Regulation of this rotational catalysis is the result of a reversible V_1_V_o_-domain dissociation that is required to preserve ATP during instances of cellular starvation. Recently the method by which the free V_1_-ATPase abrogates the hydrolytic breakdown of ATP upon dissociating from the membrane has become increasingly clear. In this instance the central stalk subunit F adopts an extended conformation to engage in a bridging interaction tethering the rotor and stator components together. However, the architecture by which this mechanism is stabilized has remained ambiguous despite previous work. In an effort to elucidate the method by which the rotational catalysis is maintained, the architecture of the peripheral stalks and their respective binding interactions was investigated using cryo-electron microscopy. In addition to confirming the bridging interaction exuded by subunit F for the first time in a eukaryotic V-ATPase, subunits C and H are seen interacting with one another in a tight interaction that provides a base for the three EG peripheral stalks. The formation of a CE_3_G_3_H sub-assembly appears to be unique to the dissociated V-ATPase and highlights the stator architecture in addition to revealing a possible intermediate in the assembly mechanism of the free V_1_-ATPase.

## Introduction

Vacuolar ATPases (V-ATPases) are biological rotary motors that harness the energy derived from ATP hydrolysis to drive the translocation of protons across a membrane. These proton pumps generate electrochemical gradients across organelle and plasma membranes to facilitate a number of secondary transport systems that are involved in a wide variety of biological processes [1]. V-ATPases are found in particularly high concentrations in many intracellular compartments such as vacuoles, endosomes, lysosomes, clathrin-coated vesicles and synaptic vesicles. Here they assist in receptor-mediated endocytosis, intracellular trafficking, apoptosis, and the uptake and storage of neurotransmitters, respectively [2]–[6]. Defects in the human V-ATPase enzyme play a putative role in number of pathologies including osteopetrosis, osteoporosis, gastritis, diabetes and cancer [7]–[10].

Structurally the yeast V-ATPase is a large complex that is composed of 14 different subunits arranged into two functional domains; a cytosolic V_1_ and a membrane-bound V_O_. The soluble V_1_-domain has a molecular mass of approximately 640 kDa and is composed of eight subunits denoted A-H that are architecturally arranged into sub-complexes according to their distinct roles in the rotary mechanism. For example, subunits A and B form the A_3_B_3_ catalytic complex that is responsible for hydrolyzing ATP and inducing the rotation of the DF central rotor stalk.

The architecture of the yeast V_1_-ATPase has been previously investigated by X-ray scattering [11], electrospray ionization-mass spectrometry [12], and electron microscopy [13]–[16], revealing the existence of three peripheral stalks. Additionally, recent work has shown subunits C and H to be positioned at the V_1_V_O_-interface [14], [17], where they interact with the three EG peripheral stalk heterodimers [14], [16], [18]. Subunits C and H are believed to undergo a conformation change that plays a major role in the regulatory dissociation process of the V-ATPase [16], [18], [19]. Together these studies indicate that subunits C and H function independently of each other however, the extent of their interactions at the V_1_V_O_-interface has remained unclear.

In the work presented here, cryo-electron microscopy (cryo-EM) was used to visualize the yeast V_1_-ATPase, revealing subunits C and H engaged in a unique interaction. Our CE_3_G_3_H reconstruction is highlighted by three distinct peripheral stalk densities that are stabilized by a CH subunit peripheral stalk base. Furthermore, these results provide evidence for the possible involvement of subunits C and H in the formation of a novel sub-complex that can assemble as part of the free V_1_-ATPase.

## Results

### Purification of Yeast V_1_-ATPase

**Figure 1:**
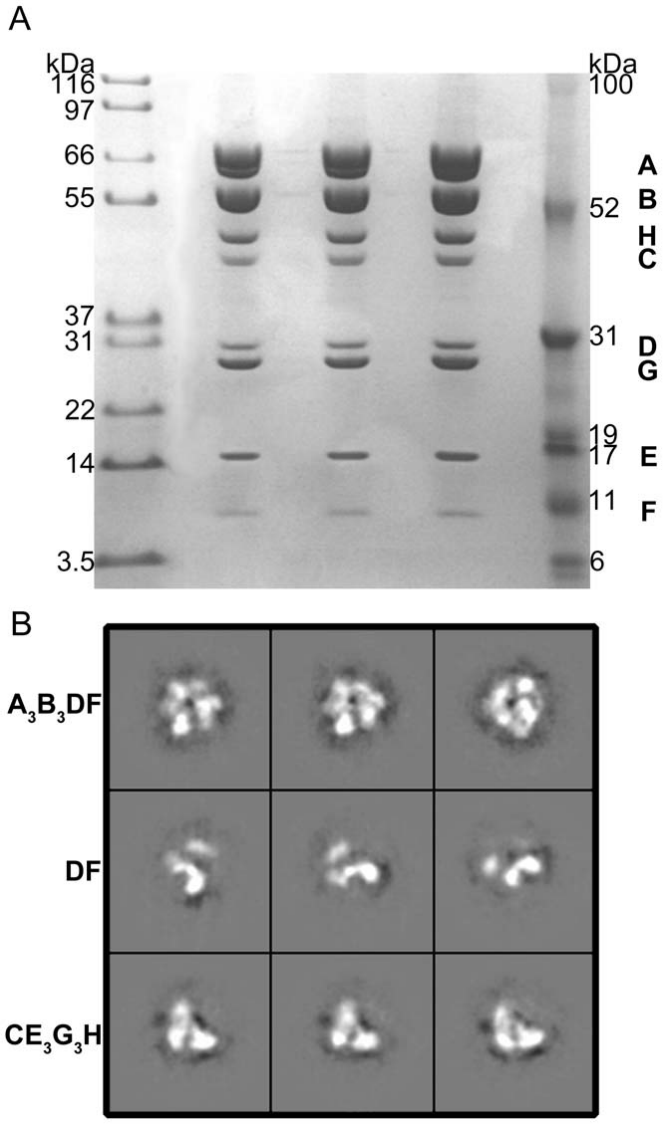
V_1_-ATPase sample homogeneity and sub-complex formation. (A) The purified yeast V_1_-ATPase as analyzed by 10% SDS-PAGE. The sample was run in three separate wells to further validate of its homogeneity. (B) The reference-free class averages of the V_1_-ATPase sub-complexes of raw particles from the cryo-EM data set. The A_3_B_3_DF complex (top row) is highlighted by a noticeable protrusion corresponding to the DF central stalk. The DF complex (middle row) assumes a boot-like overall shape and is surrounded by additional peripheral densities. The saddle-shaped CE_3_G_3_H sub-assembly (bottom row) contains strong density at the three vertices where the three peripheral stalks are bound to the C and H subunits.

Efficient isolation of the highly purified yeast V_1_-ATPase resulted from the attachment of a FLAG epitope to the carboxy-terminus of the A subunits. Subsequent purification using a FLAG antibody conjugated to an agarose resin and size exclusion chromatography resulted in a highly purified yeast V_1_-ATPase domain (Figure 1A). Detergent was not used in the purification protocol in order to avoid co-purification of the V_o_-domain with the desired V_1_ component. The fully purified V_1_-ATPase was found in a series of sub-complexes, giving rise to the discovery of the CE_3_G_3_H sub-assembly. Mechanical dissociation of the individual sub-complexes from the complete V_1_-domain may have occurred during sample purification through the size-exclusion column or may have resulted from the freeze/thaw cycles during storage of the sample prior to data collection. The identity and the molecular mass of each of the subunits A, B, C, D, E, F, G and H were experimentally determined to be 67.7, 57.6, 44.2, 29.2, 26.5, 13.4, 12.7 and 54.4 kDa, respectively using MALDI-TOF mass spectrometry.

### Three-Dimensional Reconstructions of the Yeast V_1_-ATPase

In examining the cryo-EM micrographs and the resulting class averages, it was determined that the particles existed in a number of different shapes that were inconsistent with one another. Visual identification and separation of the different particles was too difficult to accomplish manually due to the poor contrast of the cryo-EM micrographs. However, the MULTIREFINE command within EMAN [20] was able to split the data set into separate subdirectories according to particle shape (Figure 1B) (Figure S1). This separation resulted in three independent reconstructions of the A_3_B_3_DF, DF and CE_3_G_3_H sub-complexes refined to 14, 18 and 16 Å resolution respectively (Figure S2). Interestingly, the DF central stalk was captured in two separate reconstructions and in two different conformations. Collectively the three reconstructions presented here are in agreement with the most current stoichiometry of the V_1_-ATPase [12] and constitute the entire V_1_-domain.

### A_3_B_3_DF Reconstruction

**Figure 2:**
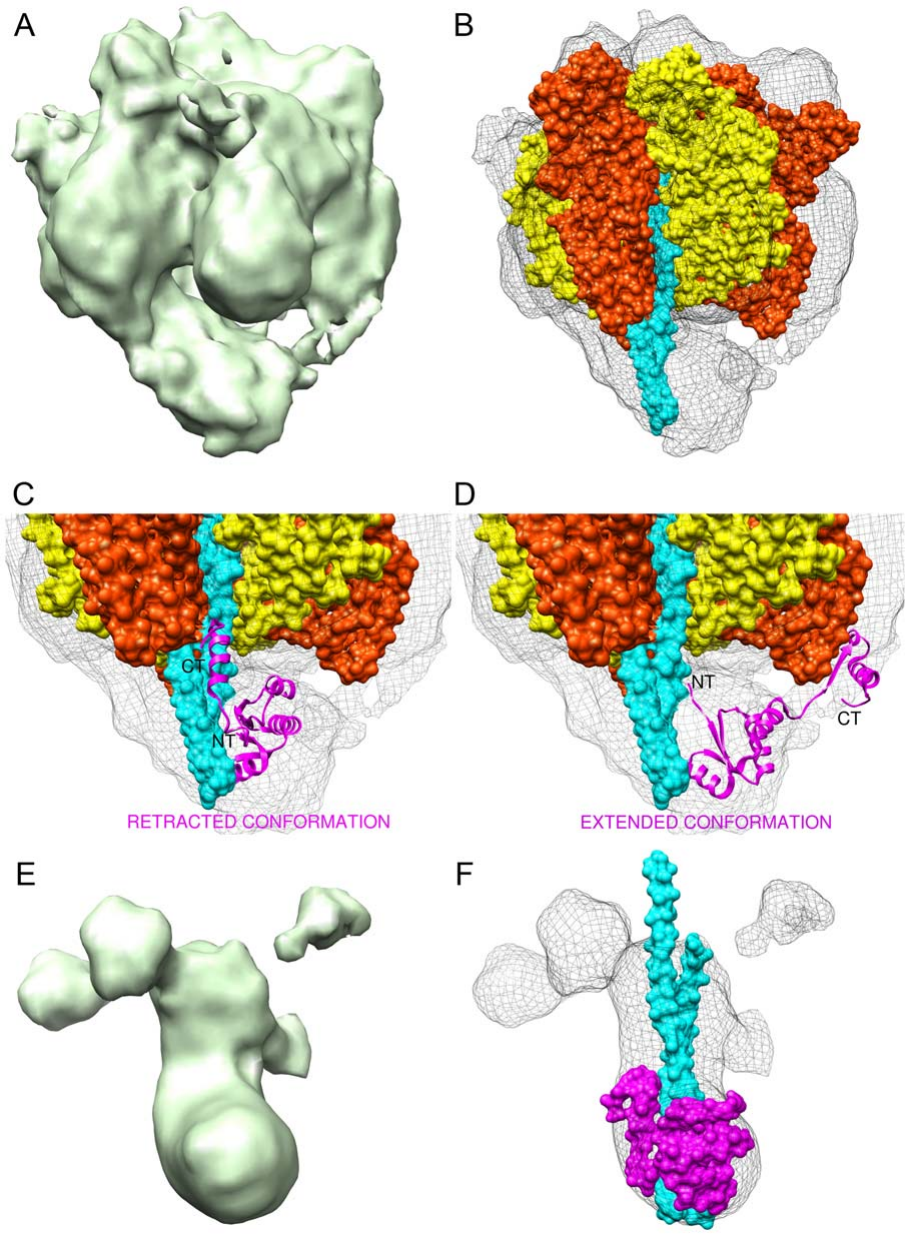
The V_1_-ATPase A_3_B_3_DF and DF sub-complexes. (A) The 14 Å resolution cryo-EM reconstruction of the catalytic A_3_B_3_DF complex. (B) X-ray coordinates for the *T. thermophilus* A_3_B_3_DF sub-assembly (PDB 3A5D) were fit into the EM density to characterize the A (orange) and B (yellow) trimers as well as the central stalk (subunit D-cyan). (C) A close up view of the central stalk interactions in which subunit F (magenta) is in the retracted conformation (PDB 3A5D). (D) The extended conformation of subunit F (magenta) from *T. thermophilus* (PDB 2D00) was fit into the central stalk density to occupy the narrow bridging interaction that tethers the central stalk to a contralateral A subunit. (E) The DF reconstruction highlighted by a boot-like structure with additional peripheral densities. (F) Subunits D and F fit into the DF reconstruction to characterize the respective subunit domains. The retracted conformation of F was fit to compliment the boundaries of the EM density.

The A_3_B_3_DF reconstruction was refined to 14 Å resolution and reveals a unique central stalk interaction that has previously only been seen in prokaryotic models (Figure 2A). At the current resolution, the six individual subunits can be discerned each having an approximate width of 35 Å. The identity of the A and B subunit locations was revealed by an automated rigid body fitting of available X-ray coordinates for the A_3_B_3_DF sub-assembly (PDB 3A5D) (Figure 2B). The three A subunits appear to have regions of their density protruding away from the center of the A_3_B_3_ complex forming knob-like densities that are thought to be representative of an amino acid insertion near the amino-termini [21]. Additionally, the three B subunits appear consistent in their shape and relative size, collectively forming a triangular cap at the point furthest away from the membrane.

The central stalk density has an approximate width of 30 Å and projects roughly 70 Å from the base of the A_3_B_3_ catalytic complex. At its insertion point into the A_3_B_3_ complex, the central stalk interacts with a single catalytic A subunit, consistent with the well-established mechanism that describes the coupling of proton-translocation and central stalk rotation to ATP synthesis in the F-ATP synthase [22]. The spatial occupancy of this density was validated with the X-ray coordinates for subunit D (PDB 3A5D) in which the long slender α-helical domain of subunit D was fit asymmetrically nearest one of the catalytic AB interfaces (Figure 2C). The central stalk also contains an additional density at its distal end corresponding to an extended conformation of subunit F. When fit with available X-ray coordinates (PDB 2D00) it was confirmed that subunit F extends contralaterally to interact with a single A subunit thus bridging the distal end of the central stalk to the base of the A_3_B_3_ complex (Figure 2D). This DFA rotor-stator interaction is also the likely cause of the central stalk bending to a 45°angle.

### DF Reconstruction

An 18 Å reconstruction of the isolated DF rotary subunits resulted from a particle alignment centered on the central stalk and not the catalytic subunits as seen in the A_3_B_3_DF reconstruction. As a result, most of the A and B trimer densities were averaged away due to their misalignment in relation to the now anchored central stalk. Four globular densities are seen above noise level surrounding the central stalk nearest its likely insertion point into the A_3_B_3_ complex (Figure 2E). The centers of three of these satellite densities are spaced roughly 70 Å apart, corresponding to the relative distance between the centers of the A or B subunits in the A_3_B_3_DF reconstruction. These densities are approximately 25 Å wide along the short axis of the V_1_-ATPase and are representative of the A or B subunits that were mostly averaged away.

The DF reconstruction features a strong tubular-shaped density that spans approximately 80 Å about the long axis of the V_1_-ATPase and is representative of subunit D as indicated by the fitting of the *T. thermophilus* coordinates (PDB 3A5D) (Figure 2F). Near its projected insertion point into the A_3_B_3_ complex this density has an approximate width of 30 Å, corresponding to the girth of the central stalk in the A_3_B_3_DF reconstruction. At the distal end, the central stalk contains a protuberance roughly 25 Å long and perpendicular to the orientation of subunit D. This feature adds to the asymmetry of the complex, giving it a “foot-like” appearance that is occupied by of the retracted conformation of subunit F. The extended conformation was unable to fit into the DF reconstruction due to a lack of density available for the carboxy-terminus to extend from the amino-terminus. These results further indicate that subunit F is in the retracted conformation in the isolated DF reconstruction.

### CE_3_G_3_H Reconstruction

**Figure 3:**
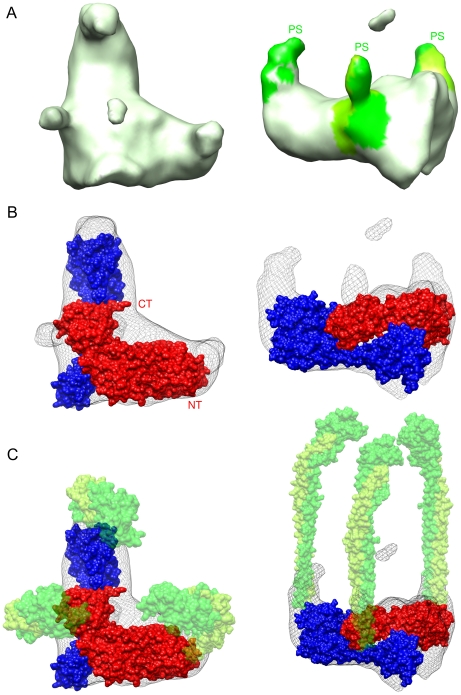
The yeast V_1_-ATPase CE_3_G_3_H Reconstruction. (A) Top and side views of the CE_3_G_3_H peripheral stalk complex characterized by the three elongated peripheral stalks. From the top of the complex viewing in the direction of the membrane, the heterodimeric peripheral stalk densities (PS) are outlined in green and lime green to highlight the likely subunit boundaries of subunits E and G. The density was colored on the basis of X-ray fitting results of the E/G complex from T. thermophilus (PDB 3K5B) with a color probing radius of 7 Å. (B) Top and side views of the subunit C (blue) and H (red) interaction highlighted by a precise inter-subunit overlap. Viewing in the direction of the membrane, the carboxy-terminus (CT) of subunit H (PDB 1H08) fits over the neck domain of subunit C (PDB 1U7L) characterizing a contact interface area of approximately 4500 Å^2^. (C) Top and side views of the CE_3_G_3_H sub-assembly with 50% transparent EG heterodimer densities (PDB 3K5B green and lime green) incorporated into the density map to further outline the orientation of the three peripheral stalks relative to subunits C and H.

The 16 Å reconstruction of the CE_3_G_3_H sub-complex contains several unique structural features that support its function and location in the intact V-ATPase (Figure 3A). The X-ray coordinates of the yeast V-ATPase subunits C (PDB 1U7L) and H (PDB 1H08) were fit into the density map to outline the subunit boundaries within the reconstruction (Figure 3B). The EMFIT [23] and COLORES [24], [25] programs were used to fit the X-ray coordinates of subunit C into the CE_3_G_3_H density based on model correlation. The ability of subunit C to bind with two peripheral stalks was also used as a determining factor in accurately docking the respective X-ray coordinates [26]. Alternative computational and manual fittings of subunit C resulted in a lack of reasonable density to accommodate the docking of subunit H. The X-ray structure of subunit H was fit in the remaining density based on recent biochemical findings in *Neurospora crassa* indicating that the amino-terminus of subunit H accommodates the binding of one of the three peripheral stalks [27]. EMFIT was used to assign quantitative values to the relative accuracy of these X-ray coordinate fitting, yielding sumf values of 52 and 60 respectively [23]. These values correspond to a high average value of density at all of the atomic positions (sumf) as a result of a well placed fit. Additionally, the percentage of atoms in negative density was 0.05 and 0.03 respectively.

The architecture of the reconstruction can be described as “saddle-shaped”, with a flat central portion and a pronounced ridge. This saddle-like ridge feature contributes to the asymmetry of the model and is likely occupied by the amino terminus of subunit H (Figure 3B). Additionally, the carboxy-terminus (residues 371–421) of subunit H was fit near residues 146–161 and 272–287 of the α-helical “neck” domain in subunit C. This CH interface has a relative contact area of approximately 4,500 Å^2^, as determined and confirmed by the CCP4 programs AREAIMOL and SURFACE [28] and the INTERSURF command within the Chimera software suite [29]. Additionally, the CE_3_G_3_H complex also contains three distinct vertices, with bulging densities below the equator of the complex protruding some 15 Å in the direction of the membrane. Two of these formations are occupied by the “head” (residues 166–263) and a “foot” (residues 1–48 and 324–373) [30] domains of subunit C, while the third is populated by the amino-termini of subunit H which also serves as the attachment point for one of the peripheral stalks.

Located in the flat central portion of the reconstruction is a shallow cavity that indents approximately 10 Å from the outer border of the complex and is roughly 20 Å wide. Surrounding this cavity are three strong slender elongated densities seen protruding in the direction of the catalytic complex that clearly represent the peripheral stalks (Figure 3A-right). These extensions are approximately 40 Å long but are likely much longer. However due to their flexibility and subsequent disorder, portions near their distal ends were averaged away. Despite the lack of rigidity, the remainder of these elongated densities could be interpreted by the fitting of the X-ray coordinates of the EG heterodimer (PDB 3K5B) (Figure 3C) [31]. It is worth noting that the presence of these three densities is not the result of particle misalignment due to the pronounced asymmetries within the reconstruction such as the large saddle-like ridge and the central cavity. Misalignment of particles would not result in such strong asymmetric features.

## Discussion

The V-ATPase is a dynamic rotary enzyme that hydrolyzes ATP through a series of differing conformational states, making it difficult to refine a three-dimensional reconstruction of the enzyme to a reasonable resolution. Additionally the fragility of the intact V-ATPase and the difficulties associated with capturing complete V-ATPase particles in a micrograph setting, contribute to the problem of acquiring a high resolution reconstruction. However, in this study the yeast V_1_-ATPase was visualized to 14 Å resolution, highlighting a novel method for the regulation of catalysis upon detachment from the membrane.

In all V-ATPases the catalytic activity arises from conformational changes resulting from ATP-binding and hydrolysis on the A subunits near the AB interface [32], [33], [34].These changes drive the rotation of the heterodimeric DF central stalk which ultimately results in the translocation of protons across the membrane through the a(cc′)_4-5_c″d proteolipid ring [35], [36], [37]. The dynamic nature of these subunits allows them to assume random orientations, resulting in their density likely being averaged away when computationally analyzed. The only situation in which the DF central stalk and the A_3_B_3_ catalytic complex could be captured in a single predominant orientation is when the two entities are held motionless by some coherent interaction as seen in our A_3_B_3_DF reconstruction. The importance of such an interaction lies in the maintenance of cell viability. During brief periods of glucose deprivation, the V-ATPase is regulated by the V_1_-domain disassembly from the membrane-bound V_O_-domain [38]. However without some form of regulation, the released V_1_-ATPase can potentially hydrolyze ATP uncontrollably. Kane and Smardon (2003) postulate that potential hydrolysis of ATP by the free V_1_-ATPase is terminated by subunit H bridging the rotor and stator stalks together [39]. A similar situation has also been explored with the F_1_-ATPase and an additional portion of the F_o_b subunit [40]. However in the A_3_B_3_DF reconstruction presented here, the abnormal tilting of the central rotor stalk to a 45°angle highlights the extended conformation of subunit F which bridges the rotor and stator entities together. Additionally, subunit F is also seen in its retracted conformation, further supporting the notion that subunit F can adopt two variable conformations depending on ATP availability [41]. The involvement of subunit F in the regulation of the free V_1_-ATPase was first proposed by Makyio et al, 2005 and later supported by crystallographic data of the A_3_B_3_DF sub-assembly from *T. thermophilus* [42]. This interaction has not been documented in a eukaryotic V-ATPase until now. By obstructing the rotation of the central stalk, the V_1_-ATPase A_3_B_3_ complex can stop cycling between the three nucleotide-binding states thus preventing the unwanted hydrolysis of ATP. A similar event has also been demonstrated in the F-ATP synthase in which the extended conformation of the epsilon subunit restricts the rotational kinetics of the gamma subunit, resulting in the inhibition of ATP hydrolysis [43].

Additionally, we present the first reconstruction of the complete peripheral stalk architecture of the yeast V-ATPase (subunits CE_3_G_3_H). In terms of subunit arrangement at the V_1_V_O_-interface, recent EM reconstructions of the complete yeast V-ATPase have proposed that subunit H is in close proximity to subunit C, however the two entities do not actually contact each other [16], [18]. On the contrary, it is now clear in the free V_1_-ATPase that the carboxy-terminus of subunit H interacts with the narrow neck domain of subunit C. This arrangement of subunits C and H appears to be extremely stable in which the interface between the two is approximately 4500 Å^2^. Collectively, the aforementioned variances observed in the subunit orientations at the V_1_V_O_-interface are supported by mutagenesis studies which suggest that the carboxy-terminus of subunit H is able to assume varying conformations depending on its involvement in either the free V_1_- or the complete V-ATPase [19], [44].

**Figure 4:**
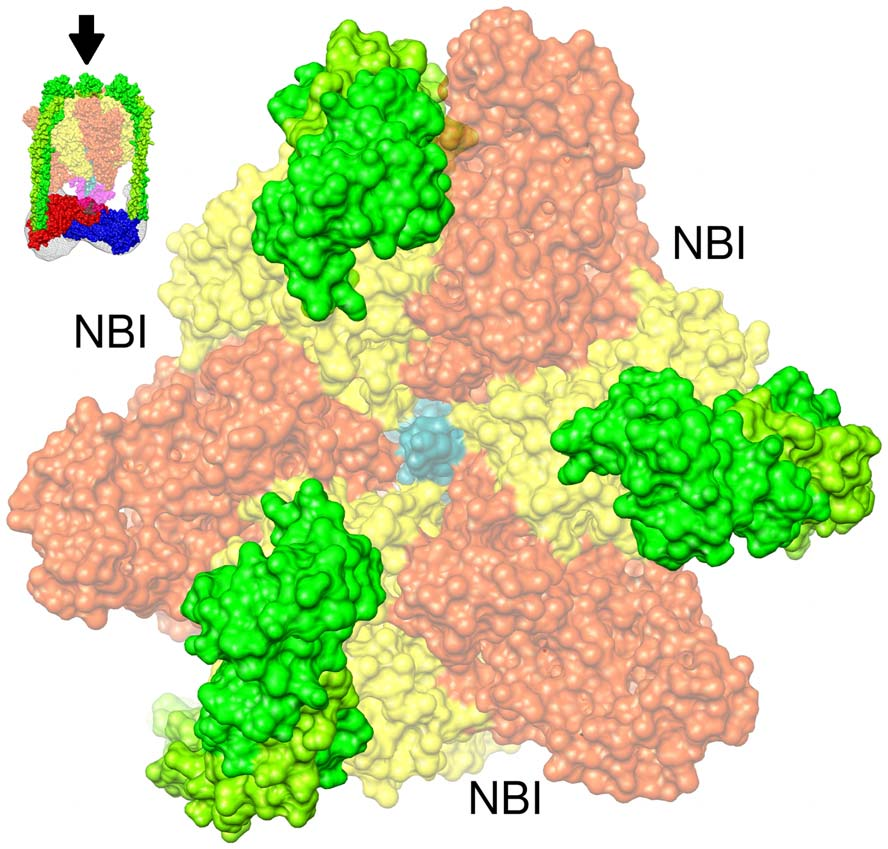
Stabilization of the catalytic interfaces. (A) A top view of the CE_3_G_3_H complex fit with a 50% transparent representation of the A_3_B_3_DF sub-assembly (PDB 3A5D). X-ray coordinates of the EG heterodimer from *T. thermophilus* (PDB 3K5B) were fit into the density map to discern the extent of the three peripheral stalk boundaries. Based on their orientation, the peripheral stalks (green and lime green) are seen interacting with the non-catalytic interfaces between the A (orange) and B (yellow) subunits [13], [17], [46]. This arrangement allows for the nucleotide-binding interfaces (NBI) to be openly accessible for binding to ATP and ADP while also facilitating enough space for rotation of the central stalk (subunit D, cyan).

The three peripheral stalks seen here are much like those considered in the V-ATPase reconstructions from the plant *Kalanchoe daigremontiana* [45], bovine [37], yeast [14], [15], [16] and the insect *Manduca sexta* [18], however in our CE_3_G_3_H reconstruction these densities are more clearly defined. Two of the peripheral stalks protrude from subunit C, one from the “head” (residues 166–263) and “foot” domains (residues 1–48 and 324–373), while the third is seen projecting from the amino-terminus of subunit H. This architecture corroborates published findings [12], [26], [27], [30]. Structurally these elongated peripheral densities extend away from the membrane and likely interact with the non-catalytic A/B interfaces [13], [17], [46] to prevent V_1_ rotation during catalysis (Figure 4). Further evidence of their stator function lies in the presence of large bulges protruding from the CH base in the direction of the membrane. The arrangement of these unique densities is such that they are likely involved in binding with the membrane-bound a subunit at the V_1_V_O_-interface [19], [26]. As a result, the EG stalks are indirectly attached to the membrane-bound stationary subunit a through their interactions with the CH base.

The physiological reasoning for the V-ATPase requiring multiple peripheral stalks lies in the arrangement of the various stator and rotor components. Based on recent X-ray [42] data, the A_3_B_3_DF complex spans an approximate distance of 170 Å down the long axis of the V_1_-ATPase. This corresponds to the distance from the top of the A_3_B_3_ catalytic headpiece to the bottom of the CH base. By comparison, the F-ATP synthase α_3_β_3_γ sub-assembly spans an approximate distance of 130 Å along the equivalent components [47]. As a result of the increased distance between the A_3_B_3_ complex and the CH peripheral stalk base, the peripheral stalks in the V-ATPase must span a longer distance compared to the F-ATP synthase therefore reducing their stability [46]. By utilizing three peripheral stalks, as opposed to one like in the mitochondrial F-ATP synthase [48], the V-ATPase is able to accommodate the larger distance and subsequently stabilize the A_3_B_3_ complex against the forces induced by the rotor.

**Figure 5:**
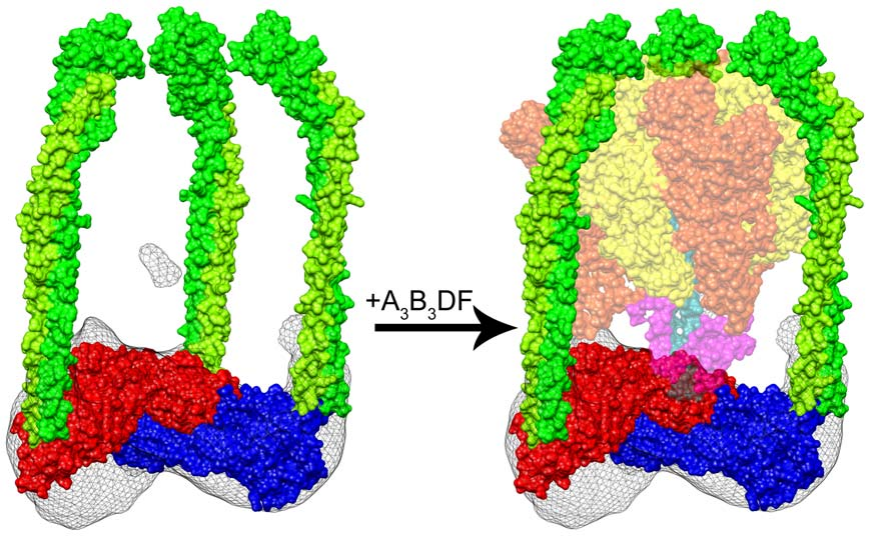
Assembly/Disassembly of the free V_1_-ATPase. (Left) Side view of the CE_3_G_3_H reconstruction fit with the subunits C (PDB 1U7L), H (PDB 1H08) and three copies of the EG heterodimer from *T. thermophilus* (PDB 3K5B green and lime green). (Right) The rigid body fit of the A_3_B_3_DF complex from *T. thermophilus* (PDB 3A5D-50% transparency), highlighting the precision by which the A_3_B_3_DF and CE_3_G_3_H sub-complexes fit together. In this arrangement, the A (orange) and B (yellow) trimers are cradled by the three peripheral stalks while the DF central stalk (cyan and magenta) is in line with the shallow cavity that surrounds the C-H interface.

Interestingly, the formation of the separate and distinct A_3_B_3_DF and CE_3_G_3_H sub-assemblies presented here sheds light on a potential assembly mechanism for the complete cytoplasmic domain. Based on the relative arrangement of the V_1_ components, it is likely that the catalytic complex and central stalk collectively dissociate from the CH peripheral stalk base containing the three peripheral stalks (Figure 5). This nascent schematic is further supported by recent high-resolution structural data revealing an A_3_B_3_DF complex free of subunits C, H, E and G [42]. While the exact mechanism for this is unclear, subunit C may play the key role as it is the only subunit that does not co-purify with either the V_1_ or V_O_ domain after complex disassembly [49]. Additionally, small angle X-ray scattering (SAXS) has revealed that subunit C can adopt two very dynamic conformations depending on its involvement in the holoenzyme [16]. Such a conformational change may be stimulated by the phosphorylation of subunit C [49], [50] which is believed to weaken the surface interactions between an EG-C complex formation [16]. However, the dissociation of subunit C from the EG stator does not appear to be the method by which the V_1_-ATPase regulates its assembly as three copies of the EG complex remain bound with subunits C and H when the CE_3_G_3_H complex is dissociated from the remaining V_1_- components.

It is clear that the CH peripheral stalk base is unique to the dissociated V_1_-ATPase and represents a dynamic sub-assembly that may be a primary constituent in the formation of the complete cytoplasmic domain. It is tempting to speculate over the mechanism by which the V_1_-ATPase is assembled, however further analysis is required to identify the physiological stimuli for such an event.

## Materials and Methods

### Subunit FLAG tagging

Triple-FLAG epitope tags were inserted at the carboxy-terminus of V-ATPase subunits by PCR based homologous recombination in the protease deficient yeast strain c13-ABYS86 [51] using Geneticin resistance as a selectable marker. Colonies were selected on Geneticin containing YPD agar plates. Single colonies were transferred into liquid YPD medium and subjected to western blot analysis using anti-FLAG antibody (SIGMA). Accessibility of the FLAG tags was assessed by pull down assays using anti-FLAG antibody coupled agarose beads.

### Enzyme Purification

*Saccharomyces cerevisiae* cells containing a carboxy-terminal 3xFLAG insert after the sequence for subunit A were cultured on YPD agar plates containing 20 mg/ml Geneticin. YEP media containing 2% glucose was inoculated with single colonies from the culture plates and incubated at 30°C. The cells were allowed to grow until the cells reached mid-log phase growth corresponding to an OD_600_ between 1 and 5. The cells were pelleted by centrifugation at 5,018×g then resuspended in Zymolyase buffer containing 50 mM Tris pH 8.0, 10 mM MgCl_2_, 1 M Sorbital and 30 mM DTT. After a second centrifugation step of 5 minutes at 1,500×g, the cells were resuspended in 3 volumes of Zymolyase buffer. To the suspension, Zymolyase 20T was added and the sample was shaken at 50 rpm at 30°C for 40 minutes until the cells were converted to spheroplasts. The spheroplasts were centrifuged at 1,500×g for 5 minutes at 4°C and the pellet was resuspended in ice-cold Zymolyase buffer. The spheroplasts were washed two more times and then lysed by osmotic shock in lysis buffer containing 50 mM Tris pH 8.0, 10 mM MgSO_4_, 1 mM EDTA, 10 mM potassium acetate, and 1 mM DTT with a cocktail of protease inhibitors. After a number of freeze/thaw cycles, 5 M NaCl was added to a final concentration of 100 mM. Anti-FLAG agarose slurry was added to the supernatant and placed in a disposable BioRad column. After thoroughly washing to remove unbound protein, the V_1_ was eluted with 500 µl elution buffer consisting of 10 mM Tris pH 8.0, 2% glucose, 3xFLAG peptide 5 mM ATP and 5 mM EDTA for a total of 4 elutions. The sample was then concentrated using a 100k cutoff concentrator unit and applied onto a Superose 6 size-exclusion column equilibrated with a buffer containing 10 mM Tris pH 8.0, 5 mM EDTA, 0.1% glucose, and 5 mM ATP. Sample purity was confirmed by SDS PAGE.

### Cryo-Electron Microscopy

The initial sample concentration was diluted from 12.2 mg/ml to 0.75 mg/ml and blotted onto holey carbon grids before cryogenic plunge freezing. The images were collected on a F20 transmission electron microscope operating at a voltage of 200,000. Data was collected on film at 50,000× magnification and under low dose data collection conditions.

### Image Processing and Analysis

Electron micrographs were scanned and digitized on a Nikon Super CoolScan 9000 ED scanner and binned to an effective pixel size of 2.4 Å. Particles were boxed using the automated algorithm in the program SIGNATURE [52]. Determination of defocus was accomplished with the program CTFFIND and Contrast Transform Function (CTF) correction was performed using CTFIT [53]. An Initial model of the A_3_B_3_DF reconstruction was created using the STARTCSYM function in the EMAN software suite assuming 3-fold symmetry (C3) to account for the pseudo-3-fold symmetry of the A_3_B_3_ complex [20]. Initial models of the DF and CE_3_G_3_H sub-complexes were derived from early MULTIREFINE experiments in which the particles were sorted into distinct subdirectories using multivariate statistical analysis (MSA). This was done assuming no symmetry (C1). Iterative refinement of the final three reconstructions was performed in subsequent MULTIREFINE experiments assuming C1 symmetry for each. The final A_3_B_3_DF, DF and CE_3_G_3_H reconstructions used 21,682, 14,873 and 20,128 particles respectively. The resolution of the final reconstructions was estimated based on a Fourier shell correlation (FSC) criterion of 0.5 using the EOTEST command within EMAN, which measured the degree of correlation when the data was split in half.

### X-Ray Fitting

X-ray coordinates for the *T. thermophilus* A_3_B_3_DF sub-assembly (PDB 3A5D), subunit F (PDB 2D00), and yeast subunits C (PDB 1U7L) and H (PDB 1HO8) were fit into the EM density maps using the programs COOT [54], CHIMERA [29], EMFIT [23] and COLORES [24], [25]. X-ray coordinates of the peripheral stalks from *T. thermophilus* (PDB 3K5B) were placed into the location they likely occupy in the V_1_ ATPase to put the reconstruction into context. The program EMFIT [23] was utilized to assign quantitative values proportional to the relative precision of the X-ray coordinate fittings. Outputs included an average value of density at all atomic positions between -100 and 100 (sumf) and a percentage of atoms in negative density (outside of density). All visualization was done with the programs VMD [55] and Chimera [29].

## Supporting Information

Figure S1 Raw Particle Images of the A_3_B_3_DF, DF, and CE_3_G_3_H Reconstructions.
(5.05 MB TIF)

Figure S2 Fourier Shell Correlations of the A_3_B_3_DF, DF, and CE_3_G_3_H Reconstructions. The final resolution values for the A_3_B_3_DF, DF, and CE_3_G_3_H reconstructions were determined to be 14, 18, and 16 Å respectively.
(1.56 MB TIF)
