# Supplementary figures and images for: The C-H Peripheral Stalk Base: A Novel Component in V1-ATPase Assembly
Source: PLoS One. 2010 Sep 3;5(9):e12588. doi: 10.1371/journal.pone.0012588 (PMC2933246; doi:10.1371/journal.pone.0012588)

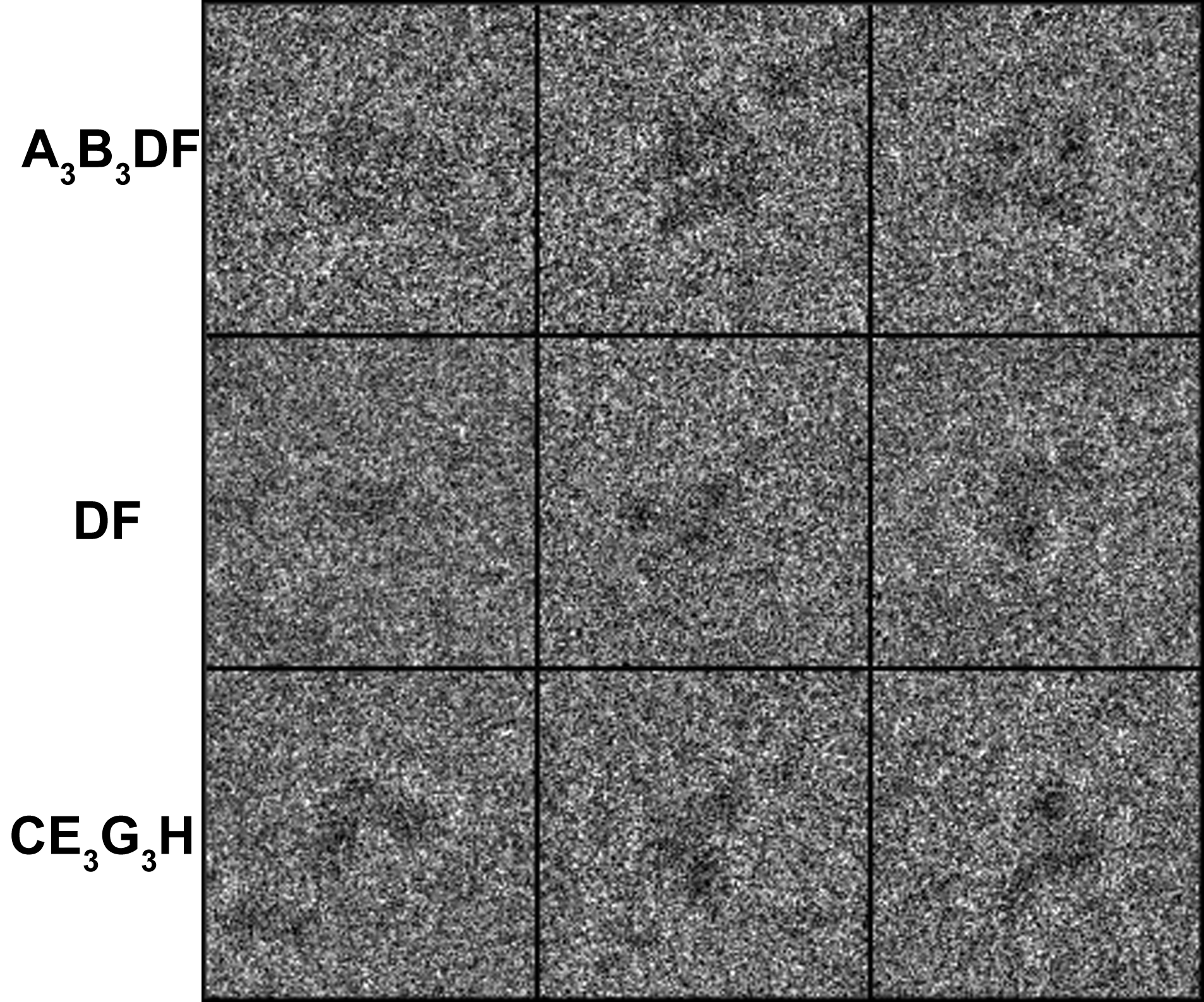

Supplement: Figure S1 — Raw Particle Images of the A3B3DF, DF, and CE3G3H Reconstructions. (5.05 MB TIF) [file pone.0012588.s001.tif]

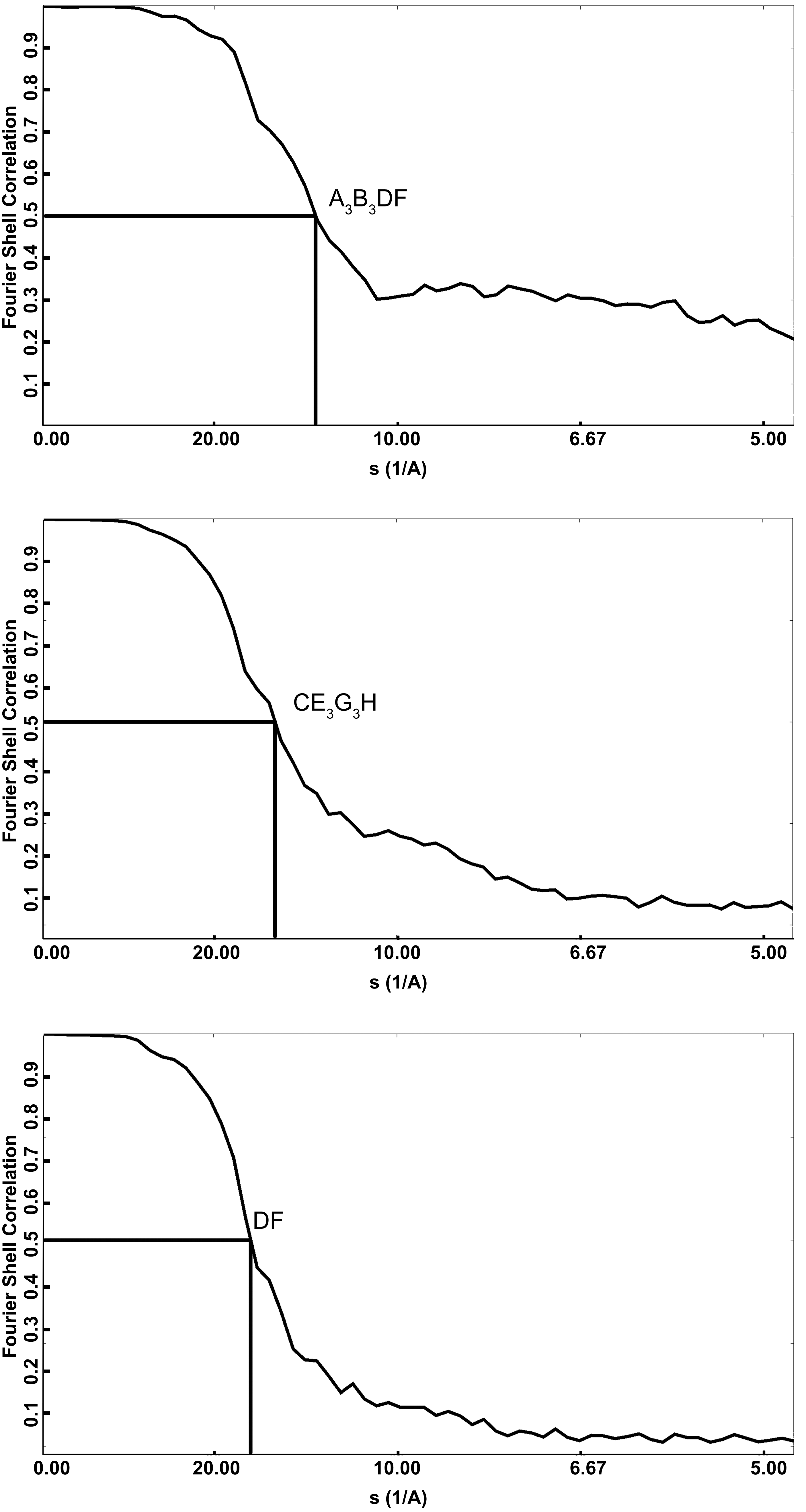

Supplement: Figure S2 — Fourier Shell Correlations of the A3B3DF, DF, and CE3G3H Reconstructions. The final resolution values for the A3B3DF, DF, and CE3G3H reconstructions were determined to be 14, 18, and 16 Å respectively. (1.56 MB TIF) [file pone.0012588.s002.tif]
